# Controlling the obesity pandemic: Geoffrey Rose revisited

**DOI:** 10.17269/s41997-022-00636-6

**Published:** 2022-04-22

**Authors:** John W. Frank

**Affiliations:** 1 Usher Institute, University of Edinburgh, Edinburgh, Scotland; 2 Dalla Lana School of Public Health, University of Toronto, Toronto, ON Canada

**Keywords:** Obesity; Causation; Primary prevention; Primordial prevention; Upstream prevention; Geoffrey Rose; Epidemiology, Obésité; causalité; prévention primaire; prévention primordiale; prévention d’amont; Geoffrey Rose; épidémiologie

## Abstract

The ongoing obesity pandemic threatens the health of hundreds of millions globally. However, to date, no country has had much success in limiting its growth, let alone reversing it. This commentary demonstrates the relevance to the obesity pandemic of the public health conceptual framework of epidemiologist Geoffrey Rose, first published as “Sick Individuals and Sick Populations” in 1985. That framework provides a useful way to analyze the pandemic’s prevention and control options, based on the notions of primordial, primary, secondary and tertiary prevention—the full spectrum of “more upstream and more downstream” approaches, each with its pros and cons. Based on an analysis of key studies to date, this commentary argues strongly that only the primordial prevention approach is likely to be successful against the obesity pandemic—but its onerous requirements for society-wide behavioural and cultural change may make that public health struggle a long one.

## Résumé

La pandémie d’obésité en cours menace la santé de centaines de millions de personnes dans le monde. Jusqu’à maintenant toutefois, aucun pays n’a eu beaucoup de succès à en limiter la croissance, et à plus forte raison à l’inverser. Notre commentaire démontre la pertinence, pour la pandémie d’obésité, du cadre conceptuel de santé publique de l’épidémiologiste Geoffrey Rose publié pour la première fois sous le titre de « Sick individuals and sick populations » en 1985. Ce cadre offre un moyen utile d’analyser les options de prévention et de contrôle des pandémies en partant des notions de la prévention primordiale, primaire, secondaire et tertiaire – tout le spectre des approches « d’amont et d’aval », chacune ayant ses avantages et ses inconvénients. Fondé sur une analyse des principales études menées jusqu’à maintenant, notre commentaire soutient fermement que seule l’approche de prévention primordiale est susceptible de fonctionner contre la pandémie d’obésité, mais que les lourds changements comportementaux et culturels que cela nécessiterait à l’échelle de la société risquent de rendre la lutte de la santé publique très longue.

## Introduction

All over the world, and especially in high- and middle-income countries, the weight-for-height of children, youth and adults is greater than ever historically recorded (Abarca-Gomez and NCD Risk Factor Collaboration 2017; Rodriguez-Martinez et al. 2020). Beginning in the United States over 40 years ago (Frank 2016), this pandemic has spread to include virtually all social classes in high-income countries (HICs), although obesity in these settings now typically exhibits the classic “gradient” in health inequality by socio-economic status seen for most common health conditions, with higher obesity prevalences among the disadvantaged (Siddiqi et al. 2015; NHS Scotland (Information and Statistics Division) 2018).

In contrast, many of the world’s low- and middle-income countries (LMICs)—particularly in sub-Saharan Africa—are still experiencing the earliest stage of the pandemic, characterized by rising levels of overweight and obesity concentrated among their most privileged sub-populations (Ellulu et al. 2014). Countries experiencing very rapid industrialization and urbanization, such as China, have seen remarkably fast—some would say “explosive”—increases in child weight-for-height within one decade (Dong et al. 2019), bringing their overall levels of overweight and obesity up to almost the same levels as the rich countries (Frank 2016).

This lagged aspect of the pandemic outside HICs means that public health and related policy and program responses to it have not been globally simultaneous. Rather, the richest nations have had decades to devise and implement effective approaches to the pandemic’s control (albeit with as yet little sustained success, particularly in terms of high-quality evidence for reducing obesity inequalities by socio-economic or ethnoracial status—Flodgren et al. 2020; Hayre 2021) whereas many LMICs have scarcely become aware of the problem and are yet to implement any potentially effective interventions against it. It is in this complex and still-evolving policy context that this essay considers the relative pros and cons of strategies for chronic disease prevention and control first enunciated by Geoffrey Rose, in his classic 1985 paper “Sick Individuals and Sick Populations” (Rose 1985). Although this landmark paper has been extensively critiqued and commented upon by others (World Health Organization 1999/2000; Frohlich and Potvin 2008), the present paper seeks to lay out in simple language and graphs just how prescient Rose was.

## Rose’s conceptual framework for prevention

**Fig. 1:**
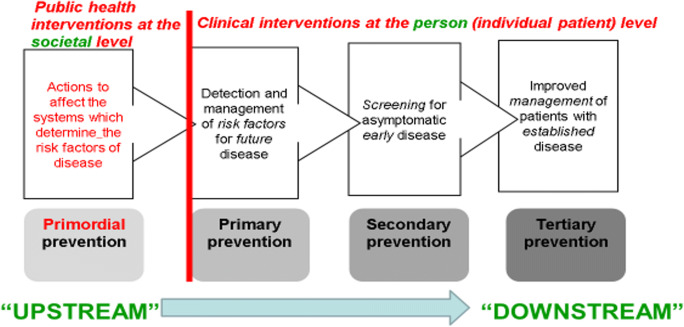
Four types of prevention spanning Rose’s two strategies (Frank et al. 2016)

Geoffrey Rose, a British cardiovascular epidemiologist, founded the Whitehall Study of English civil servants to demonstrate persistent, protean and stepwise gradients in health across civil service pay grades (Marmot and Wilkinson 2005). In Rose’s landmark 1985 paper, he lays out how chronic diseases can be tackled by two broad approaches that span “upstream”, population-wide prevention, involving public health interventions at the societal level, through to “downstream” clinical care focused on individuals at high risk—see Fig. 1 (Frank et al. 2016).

## Rose’s “Population Strategy”: interventions at the societal level

- *Primordial prevention*, a term widely attributed to Strasser (1978), was not part of Rose’s original formulation, but is easily integrated into it; it is defined as tackling the underlying reasons for the existence of chronic disease risk factors at the level of a whole society. For example, excessive salt consumption usually leads to widespread hypertension, a proven risk factor for cardiovascular disease (Rose 1985, 2008). Primordial prevention could therefore involve reducing salt in processed foods and salt use at home. However, as expanded on below, there are strong reasons to believe that such behaviourally oriented interventions would not fully reduce existing large socio-economic inequalities in salt-related health inequalities, since they would fail to tackle more fundamental drivers of many nutritionally related inequalities, such as deliberate marketing of less healthy, cheaper (but still very profitable) foods to consumers less well heeled, and often less knowledgeable about such matters (Moodie et al. 2013; Kleinert and Horton 2015). Such population-wide interventions are normally the province of public health professionals, requiring concerted advocacy to achieve government and/or industry action to change food formulation and consumption patterns. This more radical (to use Rose’s term) approach is also often termed “upstream” prevention (Dorfman and Wallack 2007).

## Rose’s “High-Risk Strategy”: interventions at the individual patient level

The following three types of more clinically oriented prevention strategies—all of them constituting inherently more “downstream” actions—focus on individuals, usually via clinical care. They were not all explicitly identified by Rose in his 1985 paper but have been extensively taught in epidemiology for decades (Porta 2008). They all suffer from what Rose pointed out is their “palliative” nature: the interventions described—unlike successful *primordial* preventive interventions—must be continued indefinitely into the future, because they do not tackle, let alone reverse, the underlying “upstream” (i.e. primordial) causes of risk-factor occurrence in the first place (Rose 1985, 2008).

- *Primary prevention*: identifying persons at high risk, who have established risk factors for the chronic disease—e.g. hypertension as a risk factor for cardiovascular disease—and medically treating those risk factors (e.g. by weight loss, dietary change, exercise, and typically long-term pharmaceutical therapy, in the case of hypertension) to reduce those persons’ future risk of adverse disease outcomes. Public health professionals may be involved in such risk-factor screening and management programs at the community level, but much of this case finding and management is done in routine primary care (note that this article, in keeping with current public health practice, considers obesity as a disease outcome, not purely as a risk factor for subsequent diseases).
- *Secondary prevention*: identifying persons with early/asymptomatic disease, through screening programs, and treating them earlier in the disease’s natural history than would otherwise be the case, in the hope of improved outcomes (survival, quality of life)—sometimes these programs are managed by public health professionals, but often they rely for delivery on primary care practitioners. A United Kingdom example is aortic aneurysm screening by ultrasound imaging, culminating in surgical referral for aneurysms large enough to be at imminent risk of complications.
- *Tertiary prevention*: diagnosing and treating persons with the fully developed disease so as to prevent recurrences and complications—e.g. after a patient’s first episode of coronary heart disease or stroke, through prescribing long-term beta-blockers, anti-platelet drugs, statins, etc.; clearly, this is a clinical approach (“chronic disease management”), dependent on integrated secondary (hospital) and primary care systems, now ethically mandated as part of high-quality care for such patients.

We now examine how these contrasting (but often complementary) approaches to chronic disease prevention could be applied to the current obesity pandemic.

## Application to the obesity pandemic

**Fig. 2:**
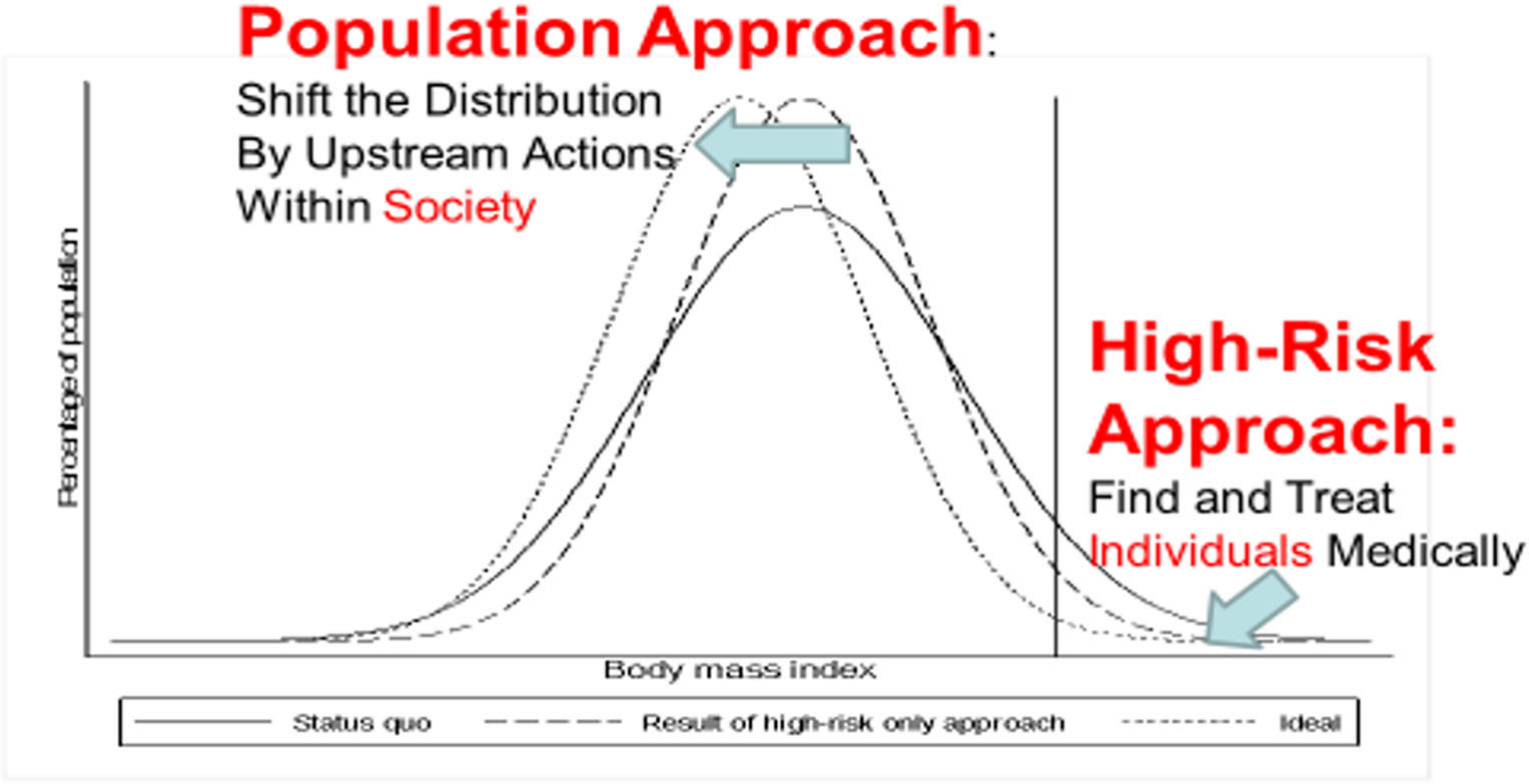
Rose’s two strategies of prevention, as applied to the obesity pandemic (Rose 1985, 2008; Frank et al. 2016)

**Fig. 3:**
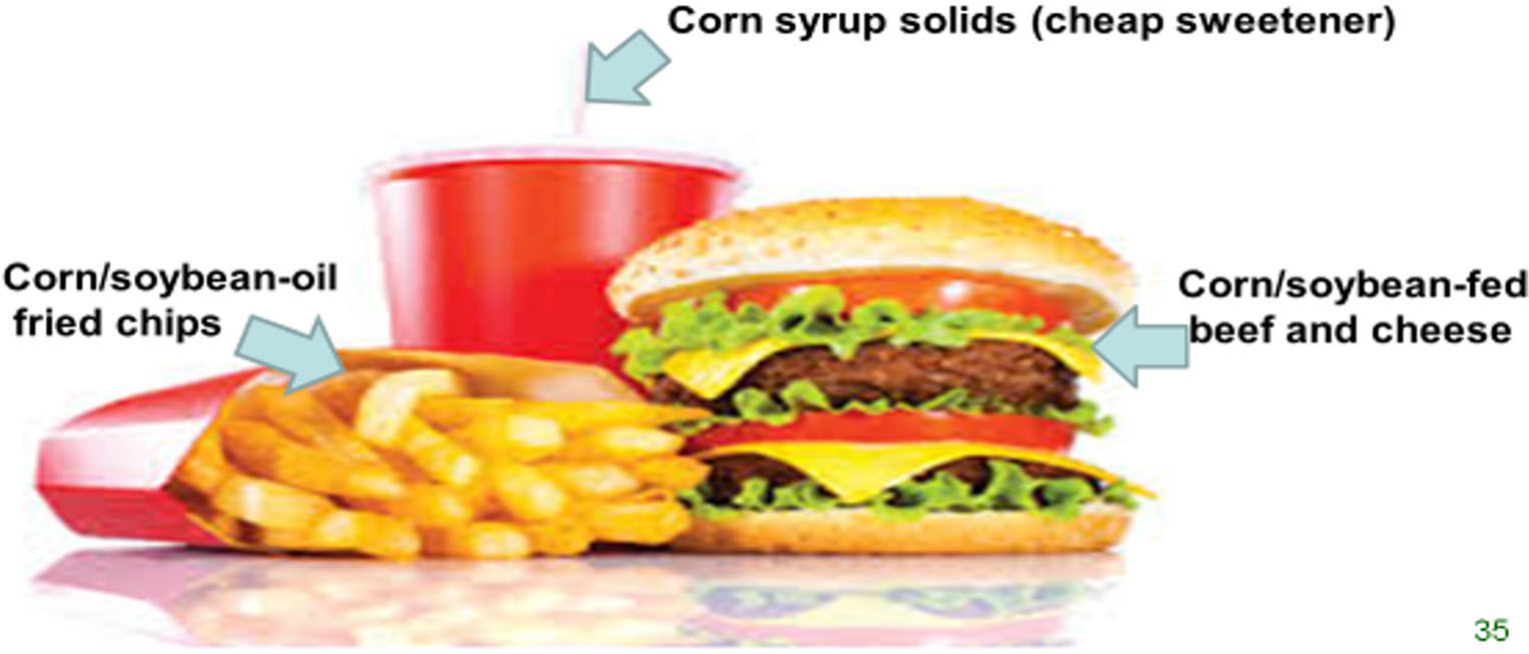
Highly industrialized nutritional components of a typical fast-food meal

The four prevention and control strategies can be applied to tackle the obesity pandemic as follows:

- *Tertiary prevention*: Find and treat medically all cases of established obesity. Virtually no society has seriously attempted this, partly due to the high costs involved, and the lack of availability/high cost of appropriately trained personnel (arguably involving an integrated team composed of physician/nurse practitioner, dietitian and psychologist). As well, because the treatments we have now are largely behavioural, they do not have a very high long-term success rate, due to patient non-compliance and drop-out (Pandita et al. 2016; Grossman et al. 2017). (This generalization does not apply to surgical treatments, such as stomach stapling, but those are only appropriate for morbidly obese patients at imminent risk of serious obesity complications.)
- *Secondary prevention*: Identify persons *at imminent risk of obesity* due to a rising BMI nearing 30 (or a lower BMI cutoff, if the patient’s overall health risks from further weight gain warrant more aggressive case finding); then treat those persons with the same sort of intensive measures described above. This strategy is potentially feasible in primary care settings in HICs, through simple regular monitoring of all enrolled patients’ weight-for-height, initiating treatment referral when patients’ BMIs reach a carefully selected “danger threshold”. However, this strategy seems never to have been formally proposed—likely because such caregivers in most HICs are already swamped by patients with full-blown obesity.
- *Primary prevention*: Identify all persons at risk of obesity in the next, say, half decade, and treat them with the same intensive measures described above. This approach, dependent on accurate multivariate prediction algorithms for future obesity based on universally available predictors, is the topic of much research in child obesity. Recent studies suggest that the best current algorithms can predict about 75% of obesity cases at age 12, based on clinical data available at school entry, including a single measurement of weight-for-height (Butler et al. 2018; Ziauddeen et al. 2018). However, a serious drawback to this approach is that it necessarily results in at least a *third* of the younger-age child population being labelled at “high risk” and referred for treatment. This is a far higher caseload than most current pediatric referral systems can accommodate for the intensive whole-family treatment programs required (Moyer et al. 2005; Butler et al. 2018; Darling et al. 2021). Such risk screening is also not necessarily a convincing investment, given the dubious evidence base thus far for the long-term effectiveness of interventions to prevent or treat child obesity (Darling et al. 2021; Nobles et al. 2021). As Rose pointed out in 1985, this strategy also flies in the face of social norms of eating and physical activity, which have now become overtly obesogenic in many countries (Gortmaker et al. 2011; Swinburn et al. 2011), thereby providing little incentive for the patient and his/her family to comply with/stay in treatment. And, of course, this approach would also have to be deployed in perpetuity, since no radical societal action to address the underlying drivers of obesity has been taken. Rose foresaw all this, as evidenced in his 1985 paper and subsequent book—see Fig. 2 (Rose 1985, 2008; Frank et al. 2016).

- *Primordial prevention*: Experts on the underlying drivers of the obesity pandemic point to the mass production and marketing of inexpensive, high-caloric-density foods, highly processed, and often designed to reduce preparation and cooking times—so-called convenience foods (Kleinert and Horton 2015; Swinburn et al. 2019). Time series of cross-national sales volumes per capita of such products, between the 1970s and 1990s (at least in the USA) could be used to provide the evidence base for this approach (Frank 2016). Support for the role of highly processed convenience foods in the pandemic comes from a remarkable sequence of events in the USA in the 1970s, where and when the pandemic appears to have begun. Eminent science journalists such as Pollan (2008) and respected researchers such as Swinburn et al. (2009) have described how US federal agricultural policy during that era massively subsidized industrial-scale cultivation in the US Midwest of corn and soy beans, followed by at-scale processing to extract low-cost and relatively unperishable nutrient components critical to convenience-food manufacturing: corn oil, flour and corn-syrup-based sweeteners, as well as soya oil and high-protein supplements for factory-farming food animals and fowl, to promote rapid growth and thus lower production costs. These low-cost inputs are essential for the mass marketing of burgers, chicken pieces, French fries (via the deep-frying oil) and corn-syrup-solids-sweetened beverages, sold at prices that typically do not include “full-ledger accounting” of the true ecological or health costs involved. Even a cursory inspection of the agricultural origins of a typical fast-food meal reveals these industrial-scale inputs required for economically competitive production (Fig. 3).

The challenge with the primordial prevention strategy, although it has the great merit of being radical (i.e. needing implementation only once for all time), is that powerful commercial, political and even cultural forces oppose it (Moodie et al. 2013; Kleinert and Horton 2015). There are strong parallels here with “upstream” strategies to control other “marketable hazards”, such as smoking and excessive alcohol use, in the face of vested commercial interests (Cantrell et al. 2013; Al-hamdani 2014; Martin-Moreno et al. 2013; Bates et al. 2018). Unfortunately, as Rose pointed out (Rose 1985), it may simply not be realistic to expect everyone in a modern democracy to profoundly change their eating habits to benefit the minority whose weight-for-height is excessive—although in some countries, such as the USA, that minority has now become a majority, with two thirds of adults either overweight or obese.

**Fig. 4:**
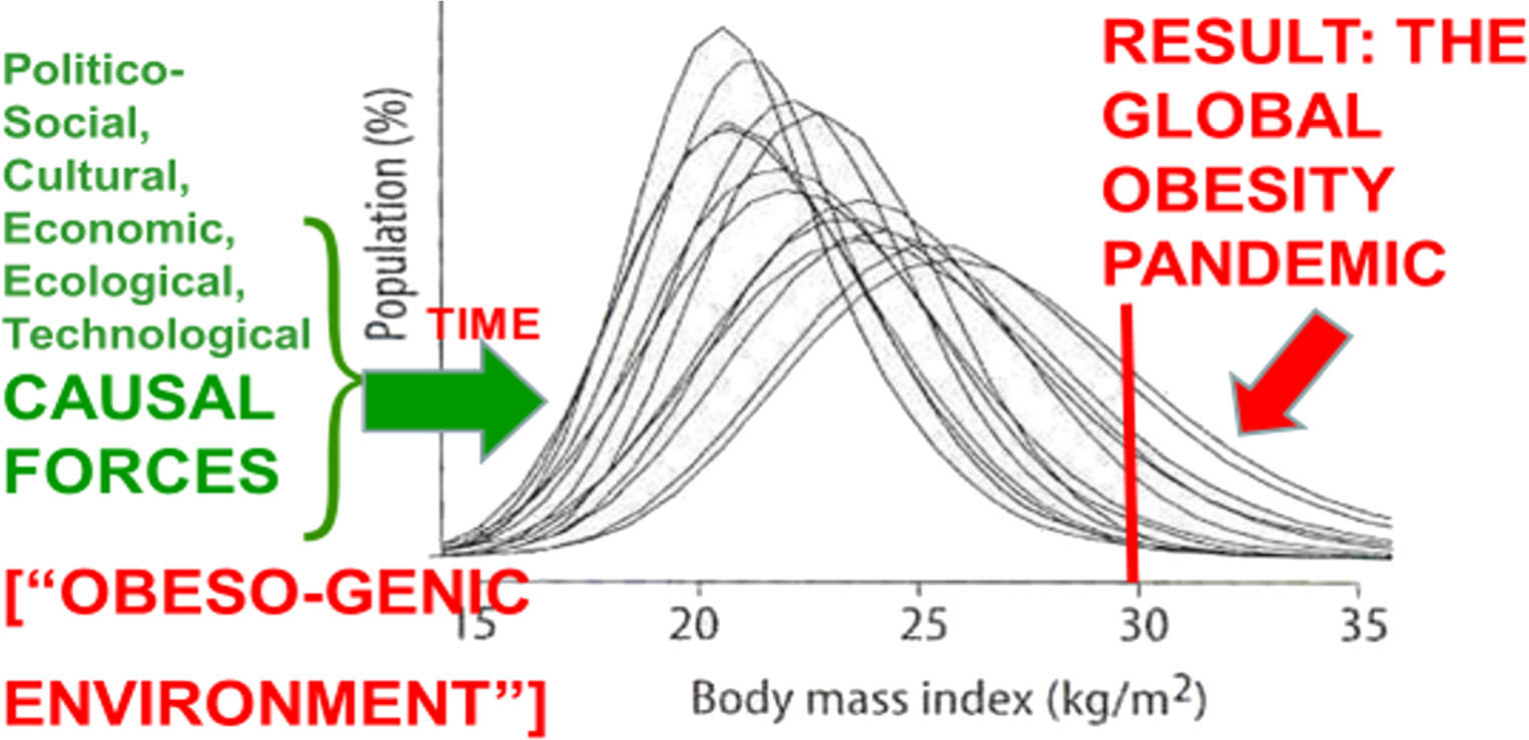
Rose’s population strategy as applied to obesity (Rose 2008)

Thirty years ago, Rose published a prescient graph (Rose 2008—first edition published in 1992) showing what the most “upstream” control option for an obesity pandemic would look like—it involves “shifting the curve” (population distribution) of weight-for-height to counter the underlying forces pushing it “to the right”—as depicted in a figure reproduced in the 2002 WHO World Health Report (World Health Organization 2002)—cf. Fig. 4, adapted from that report.

In contrast, a recent critique of a Cochrane Review of rigorously evaluated interventions to tackle the obesity pandemic found that the overwhelming majority of them inherently involved Rose’s “high-risk” approach, with—perhaps unsurprisingly—very few sustained successes demonstrated to date (Nobles et al. 2021). More positively, Crane et al. (2020) searched for “natural experimental” studies of obesity prevention, virtually all aimed at a higher-level, primordial prevention via policy interventions attempting to change dietary and/or physical activity at a community/societal level; they found 46 studies worthy of detailed review, although the authors note that all of these were published after 2007, and most after 2012, so that hardly any included follow-up for outcomes occurring more than 3 years after the interventions, leaving unanswered the question of longer-term impact. Others have gone further, calling for a sea change in obesity research, advocacy and action to move up the hierarchy of prevention, focusing on more upstream interventions at the community/societal level, to tackle the underlying drivers of the pandemic, through clearer definition of the key elements of the “obesogenic environment” those drivers have created (Gortmaker et al. 2011; Kleinert and Horton 2015; Swinburn et al. 2019).

## Conclusion

Remarkably, Rose foresaw, 37 years ago in “Sick Individuals and Sick Populations”, the precise pros and cons of the options for prevention and control of the obesity pandemic now engulfing us. Perhaps the full implementation of primordial prevention will eventually occur in the years to come, as the proportions of overweight and obese citizens, and their medically severe complications, reach levels that make matters more fully evident to the public. Were he still alive today, Rose might well simply reiterate his sage advice of 1985 to public health professionals facing the current obesity pandemic: seek the (upstream) “causes of (population) incidence, not of (individual) cases”. He surely would have spotted the rather sad truth: some pandemics simply have to get worse before difficult definitive action is taken to tackle them.

## Data Availability

N/A

## Code availability

N/A
